# King Edward's Hospital Fund for London

**Published:** 1902-03-15

**Authors:** 


### The Institutional Workshop.

### KING EDWARD'S HOSPITAL FUND FOR LONDON.

### AN ADDITIONAL TWENTY THOUSAND A YEAR FOR THE HOSPITALS.

The annual meeting of the General Council was held on the 7th inst. at York House, St. James's, H.R.H. the Prince of Wales being in the Chair. Those present were the Lord Lieutenant of London (the Duke of Fife), the Duke of Norfolk, Cardinal Vaughan, the Chief Rabbi (the Rev. Dr. Adler), Viscount Duncannon, Lord Rothschild, Lord Farquhar the Lord Mayor (Sir Joseph Dimsdale, Bart.), the Governor of the Bank of England (Mr. A. Prevost), the President of the Royal College of Surgeons (Mr. H. G. Howse), Sir Savile Crossley, Bart., M.P., Sir Trevor Lawrence, Bart., Sir John Aird, Bart., M.P., Sir Henry Burdett, K.C.B., Mr. Sydney Buxton, M.P., the Prime Warden of the Fishmongers' Company (Mr. G. F. Bodley, R.A.), Dr. W. J. Collins, Mr. J. G. Craggs, Mr. F. M. Fry, Mr. Albert G. Sandeman, and Mr. H. C. Smith.

Sir Savile Crossley having read the report of the General Council for the year ending December 31st, 1901, on the motion of H.H.R., it was received and adopted.

Lord Rothschild, in submitting the account of receipts and expenditure, and the balance-sheet for the year ending December 31st, 1901, said:—"May it please Your Royal Highness, it is my pleasant duty to submit to you the financial statement for the year ending 1901. I think, Sir, it must be a source of great satisfaction to you when you consider that this Fund, which was inaugurated by your illustrious father, His Majesty the King, has prospered and increased in popularity. I am afraid the figures which it is my duty to lay before you are perhaps in one way rather misleading, because in the amount of receipts the sums which have been contributed for the special Coronation Gift have not been included, and probably, under ordinary circumstances the amount of popularity of this Fund would be better demonstrated by those sums being included. But apart from our congratulations on the popularity of the Fund and the good work it does, I think we can congratulate ourselves upon the very small amount of money which has been spent in office expenditure. The total cost of collecting and distributing this large sum of money and of general administration is under £1,800, or less than 3½ per cent. of the money we collected. We have distributed £51,000, which was £1,000 more than the year before, and a considerable increase on the sums which we have distributed except in the first year, the inauguration year. We were enabled to distribute this £51,000 by means of the annual subscriptions which we received, amounting to about £25,000; by the donations, which were nearly £8,600; by £1,000 that we received from the trustees of the London Parochial Charities; and £7,000 that we received from the League of Mercy, which is most satisfactory; £5,000 from legacies, and £5,746 from investments. As I am on the subject of investments, I think it right to call to your notice the certificate given by the honorary auditors that they have examined the books of the Fund for the year ending December 31st, 1901, and have verified that every account is correct, subject to the market depreciation that existed in the value of the investments. I am afraid the Hospital Fund has suffered like a great many others, but it has been owing to circumstances over which we have no control, such as depreciation in gilt-edged securities and the war which is, I hope, now coming to its termination."

His Royal Highness then moved the adoption of the accounts.

In seconding the same the Duke of Norfolk said:—"I have much pleasure in seconding the adoption of this excellently drawn up account. Lord Rothschild spoke of the very cheap administration of the Fund, which would appeal to any business man. Having in view the excellent cause for which we are striving, namely, the assistance of the London hospitals, I think we ought not to be content with the continuance of the present support to the Fund, but to hope for a still larger amount being collected." The adoption of the receipts and expenditure accounts and the balance-sheet was then put and carried unanimously.

The Prince of Wales then said:—"I rejoice with you that our income for the last four years has shown a gradual and steady increase, with the result that the sum distributed this year exceeds that of the year previous by £1,000. I must say that this is most satisfactory, but at the same time our income is very far short of that hoped for by my father when he first inaugurated the Fund in 1897. I looked upon it as a very great honour that the King should have selected me to succeed him as President of this Fund, and it is needless to say that my earnest endeavour will be to carry out the work which he has initiated. With this object in view I have been considering how the permanent income of the Fund can be increased by, say, £40,000 or £50,000 a year. It sounds a very large sum, but we must not despair. Although I am aware that steps are being taken by a committee of gentlemen presided over by my friend, Lord Duncannon, who are kindly interesting themselves in the Fund to realise my object, at the same time I am anxious to take advantage of this opportunity to mention, that I have now received provisional promises of support which would from investments increase our income by £20,000 a year or nearly half the amount that we wish to secure. (Hear, hear.) It would indeed be a matter of supreme satisfaction to me if, during my first year of presidency, the income could be increased by at least £50,000 a year. I desire to convey my thanks to the General Council, the hon. treasurer, and the hon. secretaries for their services during the past year, and I have great pleasure in asking the following gentlemen if they will again grant their kind services as members of the Executive Committee, namely, Mr. Hugh C. Smith, Viscount Duncannon, Lord Rowton, Lord Rothschild, Lord Lister, Rt. Hon. C. Stuart Wortley, K.C., M.P., Sir Savile Crossley, Bart., M.P., Sir Henry Burdett, Dr. W. J. Collins, Mr. J. G. Craggs, Mr. Frederick M. Fry, Mr. William Latham, K.C." (Hear, hear.)

The Duke of Fife, in proposing a vote of thanks to His Royal Highness for presiding said: "I have to move a vote of thanks to his Royal Highness the Prince of Wales for his conduct in the chair, and I am quite sure we appreciate very much the great interest he evinces in the work of the Fund."

Cardinal Vaughan, in seconding the vote of thanks, said: "I have very great pleasure in seconding that well-deserved proposition. His Royal Highness has already won a golden certificate by the announcement he has made, and we may trust that by the end of the year his hopes will be fully accomplished." The resolution having been carried unanimously,

His Royal Highness said: "I thank you very much for the kind vote of thanks proposed by my brother-in-law and seconded by Cardinal Vaughan. I have already expressed, and it is needless for me to say it again, the very great interest I take in the welfare of this Fund. I trust before the end of this Coronation Year we shall be able to announce that the further £50,000 a year or the greater part of it has been secured, thus practically freeing the London hospitals."

The proceedings then terminated.

